# Wafer-scale two-dimensional semiconductors from printed oxide skin of liquid metals

**DOI:** 10.1038/ncomms14482

**Published:** 2017-02-17

**Authors:** Benjamin J. Carey, Jian Zhen Ou, Rhiannon M. Clark, Kyle J. Berean, Ali Zavabeti, Anthony S. R. Chesman, Salvy P. Russo, Desmond W. M. Lau, Zai-Quan Xu, Qiaoliang Bao, Omid Kavehei, Brant C. Gibson, Michael D. Dickey, Richard B. Kaner, Torben Daeneke, Kourosh Kalantar-Zadeh

**Affiliations:** 1 School of Engineering, RMIT University, Melbourne, Victoria 3001, Australia; 2 Manufacturing Business Unit, CSIRO, Clayton, Victoria 3168, Australia; 3 ARC Centre of Excellence in Exciton Science, School of Science, RMIT University, Melbourne, Victoria 3001, Australia; 4 ARC Centre for Excellence for Nanoscale BioPhotonics (CNBP), School of Science, RMIT University, Melbourne, Victoria 3001, Australia; 5 Department of Materials Science and Engineering, Monash University, Clayton, Victoria 3168, Australia; 6 Department of Chemical and Bimolecular Engineering, NC State University, Raleigh, North Carolina 27695-7905, USA; 7 Department of Chemistry & Biochemistry and Materials Science & Engineering, University of California, Los Angeles, Los Angeles, California 90095, USA

## Abstract

A variety of deposition methods for two-dimensional crystals have been demonstrated; however, their wafer-scale deposition remains a challenge. Here we introduce a technique for depositing and patterning of wafer-scale two-dimensional metal chalcogenide compounds by transforming the native interfacial metal oxide layer of low melting point metal precursors (group III and IV) in liquid form. In an oxygen-containing atmosphere, these metals establish an atomically thin oxide layer in a self-limiting reaction. The layer increases the wettability of the liquid metal placed on oxygen-terminated substrates, leaving the thin oxide layer behind. In the case of liquid gallium, the oxide skin attaches exclusively to a substrate and is then sulfurized via a relatively low temperature process. By controlling the surface chemistry of the substrate, we produce large area two-dimensional semiconducting GaS of unit cell thickness (∼1.5 nm). The presented deposition and patterning method offers great commercial potential for wafer-scale processes.

## Abstract (web summary)

One of the key challenges 2D materials still face is their uniform wafer-scale deposition. Here, the authors present a deposition method for post-transition metal dichalcogenides, based on transformation of an ultra-thin oxide layer on the surface of liquid elemental gallium onto an oxide-coated substrate.

Two-dimensional (2D) materials present many promising avenues for future technologies due to their remarkable characteristics^1^,^2^,^3^,^4^. 2D semiconductors, the most common of which are transition metal dichalcogenides, have recently attracted significant attention, particularly in electronic and optical device fabrication^1^,^3^. The initial step in fabricating such devices is the formation of the 2D sheet on a chosen substrate. Many methods have been proposed for the synthesis of 2D materials including exfoliation of flakes from a layered bulk^2^,^5^ followed by depositing the obtained flakes on a desired substrate, as well as chemical vapour deposition^6^,^7^ and atomic layer deposition^8^ techniques for directly growing 2D layers on substrates. However, large-scale, high-quality and homogeneous deposition of such 2D sheets has proven to be a major challenge. So far, only one report has addressed the wafer-scale homogeneity for the deposition of MoS_2_ using a metal–organic chemical vapour-based technique^9^. However, the temperatures used are above 550 °C that is incompatible with many electronic industry processes and, more negatively, the deposition process takes many hours that significantly adds to the cost and practicality.

Among the family of 2D materials, semiconductors based on post-transition metals of group III and VI elements have been scarcely explored. This family typically exists in the monochalcogenide form of MX, where M=Ga, In and X=S, Se, Te, with further stoichiometries based on higher oxidation states also reported^10^,^11^. A representative example of this family is gallium (II) sulfide (GaS) that has a hexagonal crystal structure with a unit cell of *a*=*b*=3.587 Å, *c=*15.492 Å (*c* is equal to two fundamental layers of GaS; Fig. 1a,b). 2D GaS has been recently explored for applications in transistors^10^, energy storage^12^, optoelectronics^13^, gas sensing^14^ and nonlinear optics^15^.

The electronic band structure of 2D GaS is particularly interesting^4^,^16^,^17^. Specially, bilayer GaS, which has a thickness equivalent to *c* of the unit cell, is an indirect bandgap semiconductor with a conduction band minimum at the M point and an associated bandgap of ∼3.1 eV and a direct transition at the Γ point with only a ∼210 meV wider bandgap^17^. Due to this modest energy difference between conduction band minimums at the M and Γ points, free carriers can be exchanged between valleys via room temperature thermal excitation^17^. Therefore, significant radiative exciton decay occurs that produces photoluminescence (PL)^14^.

Significant changes to the band structure of GaS occur when the number of layers is increased. Both direct and indirect bandgaps narrow simultaneously due to the strong interlayer interactions along the *c* axis. For bulk GaS, the bandgap is decreased by 0.4–0.6 eV in comparison to that of bilayer^10^,^16^,^18^.

Here we develop a new method for the deposition and patterning of wafer-scale 2D post-transition metal chalcogenide (PTMC) compounds. The 2D GaS deposition process described here utilizes a novel approach of placing a bulk of Ga liquid metal directly onto a silicon dioxide (SiO_2_)-coated substrate that leaves a layer of 2D oxide of gallium on the wettable areas (Fig. 1c,d). There are many properties of Ga that make it a compelling material for this process: liquid Ga has a low bulk viscosity (approximately twice that of water)^19^ that flows with ease, and unlike Hg, Ga has low toxicity and essentially no vapour pressure at room temperature^20^. Ga and Ga alloys have previously been used for printing of conducting electrical tracks;^21^,^22^,^23^ however, here instead of using the bulk metal we implement the oxide skin thatis left on the substrate. Like many post-transition metals, Ga rapidly forms a thin oxide layer on its surface when exposed to oxygen^24^. This gallium oxide layer is initially one unit cell thick^25^ that under ambient atmospheric conditions grows very slowly with time^26^. The atomically thin film is very robust and can mechanically stabilize the liquid metal against deformations. The surface oxide has been shown to adhere strongly to (wet) many materials and particularly to oxides (for example, SiO_2_)^27^. Certain surface modifications of oxide substrates, such as the functionalization with fluorocarbon chains, can inhibit this wetting effect^28^. The technique presented here is based on depositing and transforming the native interfacial metal oxide layer of metal precursors in liquid form. Group III and IV metals such as Ga, In and Sn have low melting points. It is known that in an oxygen-containing atmosphere, these metals quickly form an atomically thin protective and highly wettable oxide layer in a self-limiting reaction^24^. The presence of the oxide layer increases the adhesion of the post-transition liquid metal on oxygen terminated substrates via weak but surface dispersed Van der Waals forces^27^,^29^. After the placement of the liquid metal with its self-limiting skin onto the substrate, the metal can be removed readily due to the relatively low binding energy to its surface oxide skin that leaves the atomically thin oxide layer behind on the substrate. Harnessing these phenomena, we have devised a model process that uses low melting point Ga (29.7 °C) to deposit wafer-scale, printable 2D GaS (and also extend it to 2D indium sulfide). Here, the oxide skin of Ga are placed onto a patterned substrate that are then sulfurized via a relatively low temperature two-step process, producing large-area patterned 2D GaS. Controlling the surface chemistry of the substrate allows for selective patterning of the deposited material.

## Results

### Deposition method

**Figure 1:**
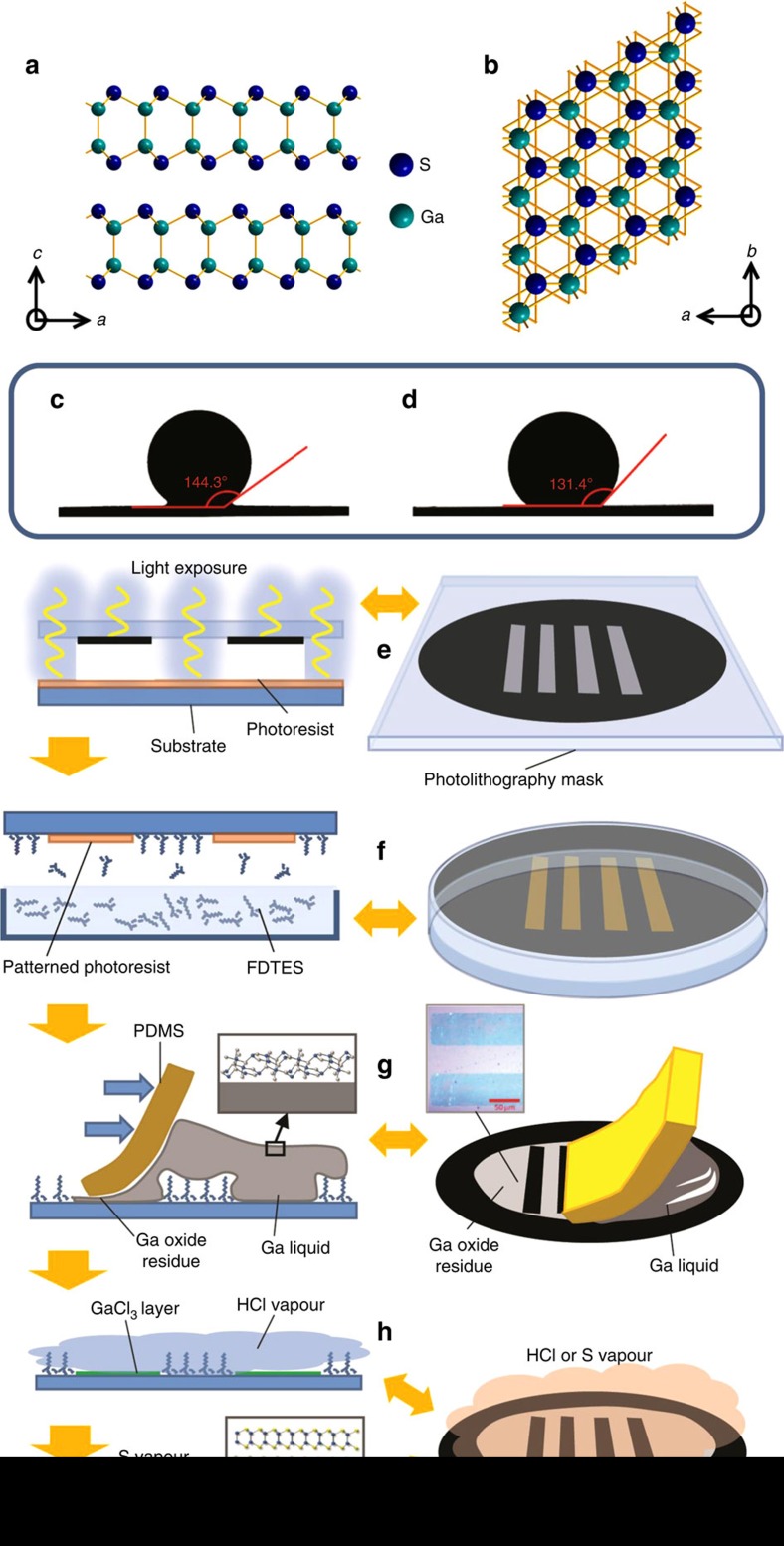
GaS representation and printing process of the 2D layers. Stick-and-ball representation of GaS crystal. (**a**) Side view of bilayer GaS, showing a unit cell *c*=15.492 Å made of two GaS layers. (**b**) Top view of the GaS crystal. The GaS crystal lattice is composed of Ga–Ga and Ga–S covalent bonds that extend in two dimensions, forming a stratified crystal made of planes that are held together by van der Waals attractions. The Ga atoms are green and the S atoms are blue. (**c**,**d**) Functionalization of the substrate with FDTES changes the contact angle between a Ga drop and the substrate by 12.9°. The SiO_2_ substrate becomes more hydrophobic and thus resists wetting by liquid Ga. (**e**,**h**) Schematics of the synthesis process for patterning 2D GaS via printing the skin oxide of liquid Ga. (**e**) Lithography process for establishing the negative pattern of the photoresist. (**f**) Covering the exposed area of the substrate with vaporized FDTES. (**g**) Placing Ga liquid metal and removing it with soft PDMS that leaves a cracked layer of Ga oxide. (**h**) Two concurrent steps of chemical vapour treatment: first, GaCl_3_ layer is formed via exposure to HCl vapour and, second, sulfurization by exposure to S vapour forming GaS.

In this work, liquid Ga is placed on a SiO_2_/Si substrate (as shown in Fig. 1e–h and described in the Methods section), leaving an atomically thin-cracked layer of gallium oxide behind, upon removal of the liquid metal (Supplementary Fig. 1 in the Supplementary Information). Selective area deposition of gallium oxide is achieved by selective treatment of the substrate using 1h–1h–2h–2h–perflourodecyltriethoxysilane (FDTES), as shown in Fig. 1e,f, resulting in nonwettable regions on the surface. The treated areas of the substrate prohibit the adhesion of the 2D gallium oxide layer (Fig. 1g), effectively facilitating selected area deposition. The printed pattern is found to be visible by eye due to interference effects (Fig. 1g inset).

A hydrochloric acid (HCl) vapour treatment (Fig. 1h) is used as an intermediary step between patterned oxide deposition and sulfurization of the oxide (see Methods section). The HCl vapour transforms the thin gallium oxide layer into primarily GaCl_3_ (refs 27, 30), aiding subsequent sulfurization via two means. First, gallium oxide is chemically inert and its direct sulfurization requires high temperatures (>900 °C) together with exposure to toxic H_2_S gas^24^. The reduced process temperature is highly advantageous since it allows the inclusion of a broad range of substrates, including certain polymers and glasses. Second, the temperature used herein during sulfurization is still higher than the melting point of the halide, thus encouraging efficient recrystallization and continuity in the resultant 2D layer. Eventually, sulfurization of GaCl_3_ (Fig. 1h) is conducted at low temperature (∼300 °C) that is compatible with existing electronic device processing standards. This process can be extended to forming other 2D PTMCs. An example for depositing atomically thin In_2_S_3_ is also presented in Supplementary Fig. 2.

### Structure and morphology

**Figure 2:**
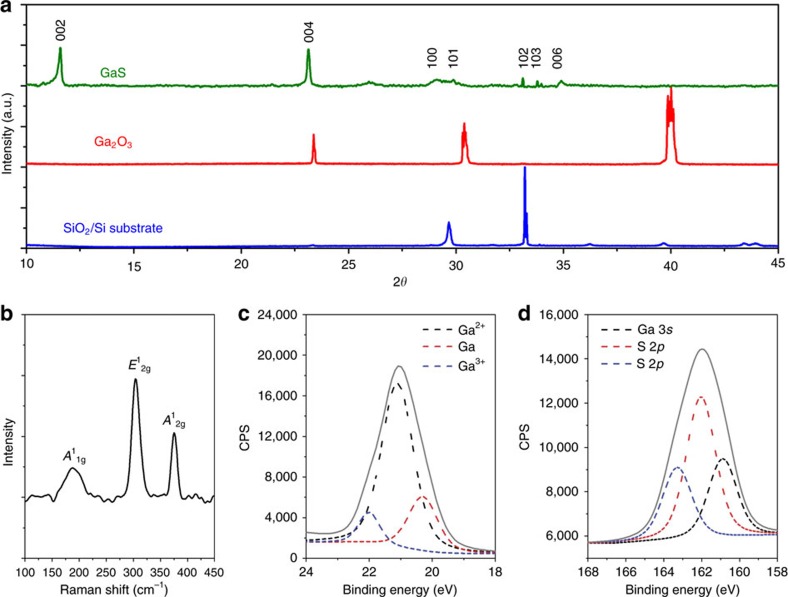
Material characterizations of printed GaS. (**a**) XRD of printed GaS film (after multiple printing steps) with XRD of the printed oxide skin and SiO_2_/Si substrate for comparison and GaS planes indicated (traces have been normalized and offset for clarity). The GaS peak corresponding to the 002 plane (11.6°) reveals a layer spacing of 7.62 Å that is in good agreement with previous studies^11^,^13^. (**b**) Raman spectrum of the 2D GaS with GaS vibrational peak shifts indicated. (**c**,**d**) XPS of the 2D GaS for the regions of interest (**c**) Ga 3*d* and (**d**) S 2*p*. A small peak from elemental Ga is also observed in the Ga 3*d* region that has been associated with the slow decomposition of GaS in moist air^11^. XPS analysis of the printed 2D GaO_x_ and GaCl_3_ are shown in Supplementary Fig. 4.

X-ray diffraction (XRD) is utilized to assess the obtained phase and phase purity of the sulfurized few-layer films (several layers of GaS are sequentially printed to increase the XRD signal intensity). The XRD patterns shown in Fig. 2a present GaS peaks, and the lack of peaks from other stoichiometries (for example, Ga_2_S_3_) demonstrate that the synthesized film has a stratified structure and the desired monosulfide phase^10^,^11^,^13^,^31^. Raman spectroscopy (Fig. 2b) conducted on a single printed film reveals the characteristic features of a few-layer thick GaS, including the *A*^1^_1g_ (187.5 cm^−1^), *A*^1^_2g_ (374.8 cm^−1^) and *E*^1^_2g_ (303.75 cm^−1^) vibrational peaks, matching those reported previously^11^,^16^. The observed relative intensity of the phonon peaks between the out-of-plane (*A*^1^_1g_, *A*^1^_2g_) and in-plane (*E*^1^_2g_) vibrations of 0.25 and 0.56 for *A*^1^_1g_/*E*^1^_2g_ and *A*^1^_2g_/*E*^1^_2g_, respectively, are uniquely associated with bilayer GaS^13^,^16^. X-ray photoelectron spectroscopy (XPS) further confirms the stoichiometry of the printed layer. The main Ga 3*d* peaks (Fig. 2c) are associated with stoichiometric GaS (Ga^2+^), with minor contributions from Ga at other oxidation states such as Ga^0^ and Ga^3+^. The S 2*p* and Ga 3*s* peaks (Fig. 2d) as well as analysis of the Cl 2*p* and O 1*s* ranges (Supplementary Fig. 3) further confirm the stoichiometry of the 2D material.

**Figure 3:**
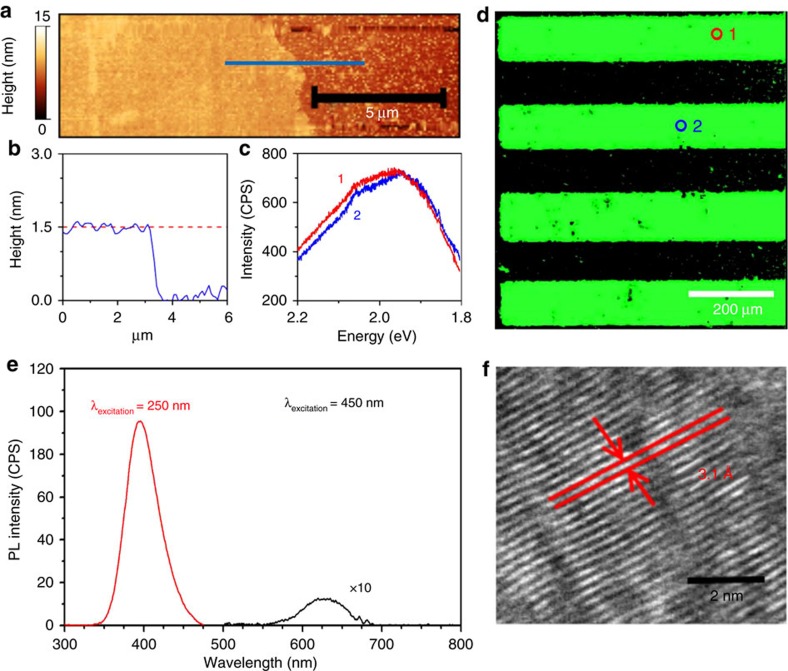
Morphology and characteristics of the printed 2D films. (**a**) AFM image with (**b**) height profile of the printed 2D GaS layer confirming bilayer deposition. The profile is offset to the substrate's surface. (**c**,**d**) Confocal PL map shows a patterned area made of 2D GaS (green) on a SiO_2_/Si substrate (black), demonstrating that a near-uniform 2D layer is obtained in a large area along with the PL spectra from areas noted on map (**c**, patterns 1 and 2). Due to the excitation wavelength limitation of confocal PL, the deep trap emission is used for assessing the uniformity of the PL pattern. (**e**) PL emission spectra demonstrating contributions of the interband transitions (red) and deep trap recombinations (black). The deep trap emission spectrum is scaled by a factor of 10 for clarity. (**f**) High-resolution transmission electron microcsopy (HRTEM) of a flake mechanically scratched from the printed GaS film. Additional HRTEM images are presented in Supplementary Fig. 5. A full discussion on continuity of the 2D films, grain boundaries and the concentration and nature of defects are presented in the Supplementary Information (Supplementary Figs 1c,d, 5 and 6).

**Figure 4:**
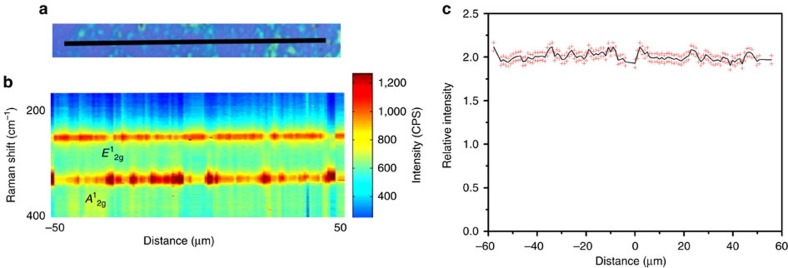
Statistical Raman analysis of the 2D GaS films. Raman line scan analysis. (**a**) Optical image, the line scan was performed on the black line indicated. (**b**) Contour plot with phonon modes indicated. (**c**) Relative intensity of the *E*^1^_2g_ phonon peak with standard error indicated. The measurements and analysis show a good uniformity for the deposited bilayer GaS. The relative intensity of the *E*^1^_2g_ peak is shown to present a standard error of only 2.5% across an area spanning 100 μm.

**Figure 5:**
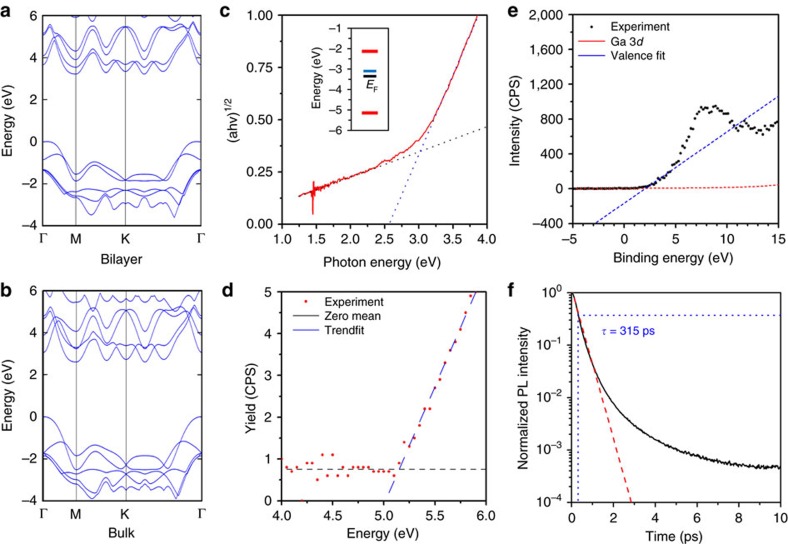
Optical and electronic properties of 2D GaS. Hybrid DFT computation of the band structures in bilayer (**a**) and bulk (**b**) GaS. For comparison, the DFT calculations for three- and four-layer GaS are presented in Supplementary Fig. 7. (**c**) Tauc plot used for determining the electronic band transition with simplified electronic band diagram (inset). Red lines are the valence and conduction band edges, black is the Fermi level and blue is the location of the deep trap band. (**d**) PESA demonstrating the valence band maximum (VBM) energy is −5.15 eV. (**e**) XPS valance analysis revealing an energy difference of 1.8 eV between the VBM and Fermi level. (**f**) PL decay with fitted exponential (red) and mean excitonic decay (blue) time indicated.

Atomic force microscopy (AFM) (Fig. 3a,b) reveals that the films have a thickness of ∼1.5 nm, corresponding to two layers of GaS, in good agreement with the Raman spectrum. The surface of the 2D film is also shown to be atomically smooth (Supplementary Fig. 1c,d). High-resolution transmission electron microcsopy (TEM) (Fig. 3f) shows a lattice spacing of 3.1 Å that correlates to the 101 plane of GaS^31^. Confocal PL microscopy (Fig. 3d) verifies that the GaS has been successfully patterned. This PL is only seen after sulfurization (Fig. 3c–e). The PL has two major components centred at 393 and 641 nm. The peak at 393 nm is due to the direct bandgap recombination at the Γ point. The energy difference associated with this peak is in excellent agreement with our density functional theory (DFT –Methods section) band structure calculations of bilayer GaS (Fig. 5a). Additionally, defects in GaS crystals are known to produce deep trap recombination states^32^ that are associated with the 641 nm peak. One of the main outcomes of this work is the deposition of patterned 2D GaS displaying near-uniform PL on a large scale with consistent PL intensity (Fig. 3c). The dimensions of the patterned area shown in Fig. 3d exceed 1 mm, proving that 2D GaS covering areas of mm^2^ can be readily achieved. A statistical analysis using Raman spectroscopy investigating the uniformity of the bilayer GaS is also in Fig. 4. The measurements and analysis show a good uniformity for the deposited bilayer GaS. The relative intensity of the *E*^1^_2g_ peak is shown to have a standard error of only 2.5% (Fig. 4c) across an area spanning 100 μm.

### Electronic and optical characterization

DFT calculations show indirect and direct recombination paths of 3.08 and 3.29 eV, respectively (Fig. 5a). The bandgap of the 2D GaS film is determined as 3.02 eV, using ultraviolet–visible absorption spectroscopy (Fig. 5c). The measured bandgap is in good agreement with the indirect transition of bilayer GaS further evidencing the near-uniform nature of the films. Significant PL is also experienced (Fig. 3e). The measured PL values (from deep trap emissions and direct recombinations) are within suitable wavelength for establishing optical devices in the middle and high end of the visible range. To obtain a more comprehensive picture of the semiconducting properties for the printed 2D GaS, the band structure of the material is analysed utilizing the PL measurements (Fig. 3e) in combination with photoelectron spectroscopy in air (PESA) and XPS valence spectra analysis (Fig. 5d,e). PESA determines a valence band maximum energy of −5.15 eV and the XPS valance spectrum determined the Fermi level to reside at −3.35 eV, leading to the energy diagram shown in Fig. 5c inset. This shows that the material is n-doped. It is also possible to obtain the p-doped areas of this 2D GaS by selectively exposing it to gases such as NO_2_ (ref. 14) to obtain the complimentary semiconductor for creating electronics. The PL decay of deep trap emissions (Fig. 5f) within 2D GaS show a mean excitonic recombination time of 315 ps that is faster than many other 2D semiconductors including 2D MoS_2_ (∼800 ps)^33^,^34^.

### Device characterization

**Figure 6:**
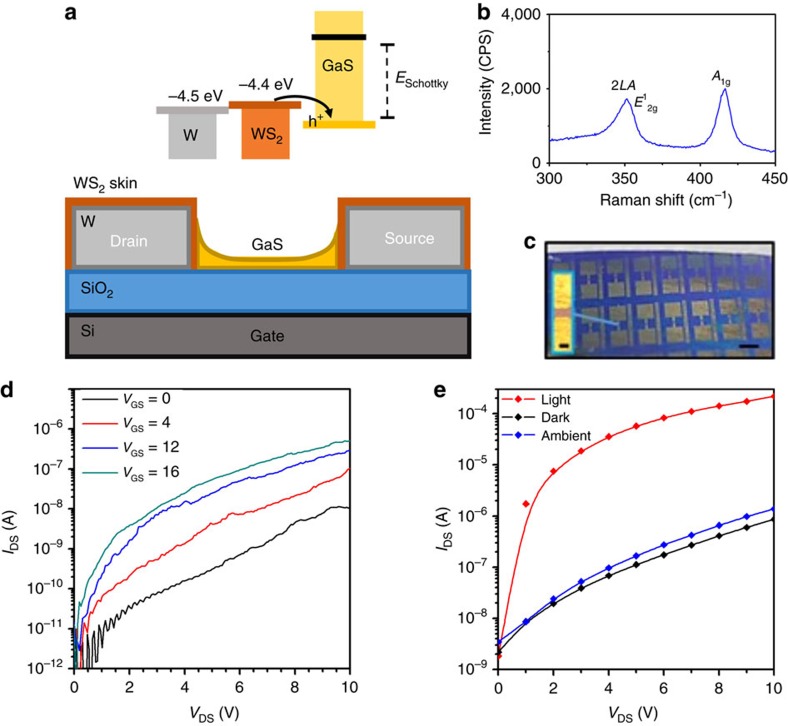
Characterization of 2D GaS FETs. (**a**) Schematic representation of back gated GaS FET with tungsten/WS_2_ electrodes featuring band energy diagram. (**b**) Raman spectrum of the W electrodes after sulfurization. Phonon modes of WS_2_ are indicated demonstrating the growth of WS_2_ on the skin of the electrodes. (**c**) Optical images of the fabricated devices and electrode gap (inset). The scale bars are 500 and 20 μm, respectively. The fabrication process is outlined in Supplementary Fig. 8. We tested five FETs at different locations and the mobility measurements have been consistent. (**d**) *I/V* characteristics of the FET at different gate voltages. (**e**) *I/V* phototransistor characteristics with no gate bias.

**Figure 7:**
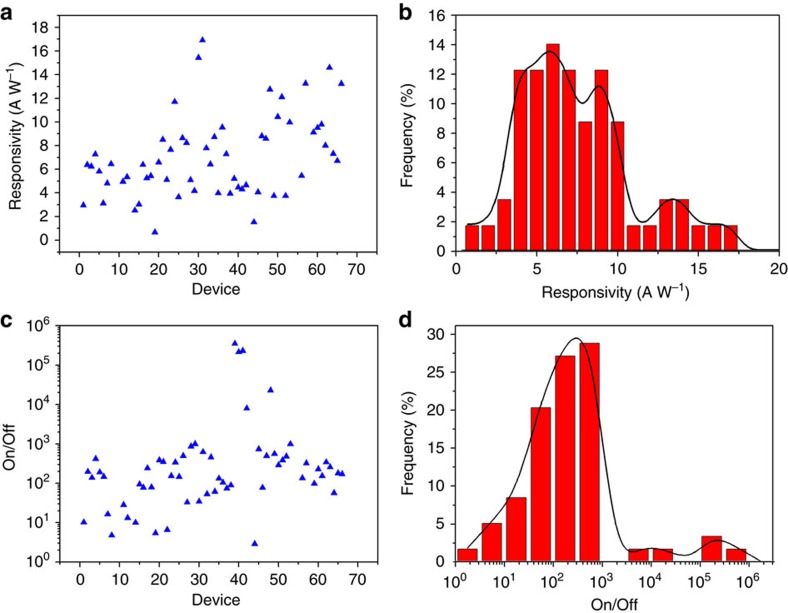
Statistical analysis of photodetector devices. (**a**) Responsivity of each individual photodetector device and (**b**) distribution of responsivities. (**c**) On/off ratio of each device and (**d**) distribution of ratios. The median responsivity is ∼6.4 A W^−1^ and the median on/off ratio is ∼170.

To investigate the potential for electronic and optoelectronic devices, field effect transistors (FETs) are developed via the deposition of tungsten (W) electrodes before deposition of Ga (Fig. 6a,c). W is chosen as an electrode material for several reasons. First, Ga does not amalgamate with W as it does with many other metals^35^. Second, W readily forms WS_2_ under the same conditions that we use for sulfurizing the gallium oxide layer (Fig. 6b). WS_2_ is known to act as a hole injector that can be used to overcome the Schottky barrier^36^. A maximum electron mobility of ∼0.2 cm^2^ V^−1^ s^−1^ is obtained that is better than previously reported mobilities for GaS^10^ and higher than that of many other printable semiconductors^37^. The on/off ratios of the FETs are above ∼150 (Fig. 6d). Furthermore, we achieve median optical on/off ratios of ∼170 in phototransistors (Fig. 6e) and a median responsivity of ∼6.4 A W^−1^ under a solar simulator that is comparable with previous reports^13^. A statistical analysis of the performance of many phototransistors on fabricated using the here reported wafer-scale process was conducted. In all, 66 devices were fabricated across two ∼1 cm × 4 cm substrates (30 and 36 devices, respectively). Of these devices, 59 were functional (27 and 32 on each substrate, respectively) resulting in an average success rate of 89.4% for the device fabrication. This distribution of responsivity and optical on/off ratio are shown in Fig. 7.

## Discussion

This paper describes a printing method that allows the selective patterning of atomically thin semiconducting layers of 2D PTMCs. The process relies on the efficient transformation of an ultra-thin oxide layer on the surface of liquid elemental gallium onto an oxide-coated substrate. Low temperature sulfurization leads to the formation of highly uniformly distributed semiconducting GaS bilayers of ∼1.5 nm thickness. There are several advantages of this method including the relative simplicity and low temperature, cost effectiveness and scalability of the process (a comparative assessment is presented in Supplementary Table 1). The method is compatible with well-established industry processes, and offers etchless patterning and deposition of 2D semiconductors on a wafer scale. Furthermore, we demonstrate the development of electronic and optical devices based on 2D GaS, with the performance better than or comparable to previous reports on exfoliated GaS layers. This process for printing 2D semiconductors, which can be extended to the deposition of other 2D PTMCs, will provide a new paradigm for future electronics, optics, optoelectronics, sensors and other devices.

This work can be possibly expanded to create other types of 2D metal compounds. Gallium can alloy a large number of different metals. As such, the self-limiting oxide layer can be tuned depending on the type and concentration of the alloyed metal, allowing the possibility of printing a large variety of pure and mixed oxides. The 2D oxides can be deposited and patterned on wafer scale and chalcogenetated. This means that gallium (or many other low melting liquid metals) can be potentially used as a reaction media to establish desired 2D metal-based compounds that should be further investigated.

## Methods

### Materials

Ga (99.99%), sulfur (S; 99.98%), HCl (36.5–38%), hydrobromic acid (HBr; 48%) and FDTES (97%) were purchased from Sigma-Aldrich. AZ-1512 photoresist, AZ-400k developer, acetone and isopropyl alcohol (IPA) were purchased from VWR chemicals. Indium (In; 99.99%) was purchased from RotoMetals. Polydimethylsiloxane (PDMS) and crosslinking agent were purchased from the Dow Corning Corporation. All chemicals were used without further processing unless otherwise stated.

### Preparation of materials

Ga was placed in a 1% v/v solution of HCl to dissolve the surface oxide. PDMS was cured with a 10:1 ratio (elastomer/crosslinker) and allowed set for a minimum of 48 h at room temperature under vacuum and then plasma treated under O_2_ flow. Wafers of 200 nm SiO_2_ on Si (SiO_2_/Si) were washed with acetone, IPA and distilled water in sequence, baked at 125 °C and cleaned with an O_2_ plasma before use.

### Synthesis of 2D GaS

The Ga in HCl was heated to 60 °C and placed onto a SiO_2_/Si substrate also heated to 60 °C as described in Fig. 1. The liquid gallium was then spread across the substrate followed by its removal via wiping using soft PDMS. The gallium oxide remains on the silicon oxide surface during the wiping process due to the very strong van der Waals adhesion between gallium oxides and the substrate. However, the liquid gallium has a much weaker adherence to the deposited gallium oxide and thus can be easily removed by wiping the excess liquid metal, leaving a thin layer of cracked gallium oxide behind. The printed wafer was then cooled to room temperature. The substrate was then held directly over warm (45 °C), fuming HCl (37%) for 5 min to achieve chlorination. The chlorinated sample was then sulfurized via a chemical vapour method in a tube furnace under N_2_ flow (∼100 cm^3^). For sulfurization, S powder was placed in a tungsten boat half way down the furnace and heated to 400 °C for 90 min, while the GaCl_3_ samples were placed face down on a tungsten boat downstream heated to ∼300 °C. 2D In_2_S_3_ was synthesized in the same manner; however, a few minor changes were applied. Indium metal was heated to 170 °C for melting the metal and during the deposition of the oxide. The oxide was then treated with HBr fumes as opposed to HCl. This acid was chosen due to the more favourable melting temperature of indium bromide that is lower than that of indium chloride. The HBr-treated sample was then sulfurized at 400 °C.

### Fabrication of structures

AZ 1512 was spin-coated onto a cleaned SiO_2_/Si wafer at a speed of 6,000 r.p.m. to achieve a thickness of ∼1 μm. The wafer was then soft baked at 95 °C for 60 s before ultraviolet exposure (350–450 nm) through a patterned photomask. The exposed wafer was then developed in a 1:4 solution of AZ 400 K and H_2_O for 45 s and then rinsed thoroughly with H_2_O. The wafer with patterned photoresist was then suspended face down on the roof of a Pyrex Petri dish containing 200 μl of FDTES. The Petri dish was placed on a 120 °C hotplate for 2 h to functionalize the exposed SiO_2_ with a monolayer of fluorocarbon. The roof of the Petri dish was then flipped and the hotplate heated to 180 °C for 30 min to evaporate any excess FDTES. The wafer was then washed with acetone, IPA and water to remove the photoresist. The patterned substrate was then printed with Ga as outlined above.

### Fabrication of transistors

Tungsten (W; 20 nm) was electron beam (e-beam) deposited onto a SiO_2_/Si wafer. AZ 5214E was then spin-coated onto the W coated wafer at a speed of 4,000 r.p.m. The wafer was then soft baked at 110 °C for 60 s before ultraviolet exposure through a patterned photomask. This step was followed by reverse baking of the wafer at 120 °C for 120 s followed by flood exposure to ultraviolet. The exposed wafer was then developed in a 1:4 solution of AZ 400 K and H_2_O for 30 s and then rinsed thoroughly with H_2_O. The substrate was then placed in 50 ml of a 30% solution of H_2_O_2_ and ∼100 mg of NaOH for 10 s to remove all unprotected W. 2D GaS was then printed as outlined above.

### Characterization

XPS was performed using a Thermo Scientific K-alpha system. The X-ray source was a monochromated Al Kα source. EDX spectra were collected using a FEI Nova nano-SEM fitted with AZTEC X-ray detector. Spectra were collected with a 3 KeV accelerating voltage chosen to provide sufficient range of measurement while attempting to limit substrate contributions. XRD was performed using a Bruker D4 Endeavour featuring a CuKα X-ray source (λ=1.54 Å). Raman measurements were conducted utilizing two systems, a WITec alpha 300R and a Horiba Scientific LabRAM HR evolution Raman spectrometer, both using a 100 × objective, and a 532 nm laser with a spot diameter of ∼500 nm was used. AFM images were collected using a Bruker Dimension Icon AFM with scan-assist software. Confocal PL maps were gathered with a Nikon N-STORM in confocal mode with excitations at 488 and 640 nm. PL and ultraviolet–visible spectroscopy were conducted on GaS printed on quartz substrates as opposed to Si/SiO_2_, and the method was otherwise unchanged. PL spectra were collected using a PerkinElmer LS50 and were averaged over 10 scans. Ultraviolet–visible measurements were performed on a PerkinElmer Lambda 1050 fitted with an integrating sphere. Additional PL measurements including decay-time measurements were conducted using on a custom build instrument using a Fianium, WhiteLase SC400-8 supercontinuum optical source delivering ∼200 μW average power to the sample with a 10 nm optical bandwidth and a 100 × air objective. A waveplate polarizer was incorporated into the excitation path to obtain polarized PL maps. Polarized maps were collected successively in increments of 5°. TEM images were taken using a JEOL 2100F STEM with a 200 kV accelerating voltage. Preparation of TEM samples involved exfoliating flakes from the printed GaS film by gently scraping with a razor blade. PESA was conducted using a Riken Keiki AC-2, photoelectron spectrometer. FET and photodetector characterizations were performed using Keithley SCS 4200 semiconductor characterization system, Keithley 2001 multimeter and ABET Technologies 150 W Xenon light source. Space-charge-limited currents were measured using a Keithley 2600 Source Meter.

### Computational analysis of band structure

Spin-dependent hybrid DFT calculations were performed using Gaussian basis set *ab initio* package CRYSTAL14 (ref. 38). The B3LYP hybrid exchange-correlation functional was used^39^, augmented with an empirical London-type correction to the energy to include dispersion contributions to the total energy. The correction term was the one proposed by Grimme^40^ and successfully used with B3LYP to calculate cohesive energies in dispersion bonded molecular crystals^41^. For all atoms, a triple zeta valence basis set, with polarization functions, was used to model the electrons^2^,^42^. A 9 × 9 × 9 Monkhorst-Pack k-point mesh^43^ was used to calculate band structure and total electronic density of states for GaS bulk, while a 9 × 9 × 1 k-point mesh was used for the monolayer, bilayer, trilayer and four-layer slabs. Each structure was each independently geometry optimized before calculation of the electronic properties.

### Data availability

The data that support the findings of this study are available from the corresponding authors upon reasonable request.

## Additional information

**How to cite this article:** Carey, B. J. *et al*. Wafer-scale two-dimensional semiconductors from printed oxide skin of liquid metals. *Nat. Commun.* **8,** 14482 doi: 10.1038/ncomms14482 (2017).

**Publisher's note**: Springer Nature remains neutral with regard to jurisdictional claims in published maps and institutional affiliations.

## Supplementary Material

Supplementary Information Supplementary Figures, Supplementary Tables and Supplementary References
