# Corrigendum: Wafer-scale two-dimensional semiconductors from printed oxide skin of liquid metals

**DOI:** 10.1038/ncomms15116

**Published:** 2017-03-22

**Authors:** Benjamin J. Carey, Jian Zhen Ou, Rhiannon M. Clark, Kyle J. Berean, Ali Zavabeti, Anthony S. R. Chesman, Salvy P. Russo, Desmond W. M. Lau, Zai-Quan Xu, Qiaoliang Bao, Omid Kavehei, Brant C. Gibson, Michael D. Dickey, Richard B. Kaner, Torben Daeneke, Kourosh Kalantar-Zadeh

Nature Communications 8: Article number: 14482; DOI: 10.1038/ncomms14482; published: 02 17 2017; Updated: 03 22 2017

The original version of this Article contained a typographical error in the spelling of the author Omid Kavehei, which was incorrectly given as Omid Kevehei. This has now been corrected in both the PDF and HTML versions of the Article.

