# Supplementary material for: Wafer-scale two-dimensional semiconductors from printed oxide skin of liquid metals
Source: Nat Commun. 2017 Feb 17;8:14482. doi: 10.1038/ncomms14482 (PMC5336573; doi:10.1038/ncomms14482)
Supplement: Supplementary Information — Supplementary Figures, Supplementary Tables and Supplementary References [file ncomms14482-s1.pdf]

Supplementary Information

Wafer Scale Two Dimensional Semiconductors from Printed Oxide Skin of Liquid Metals

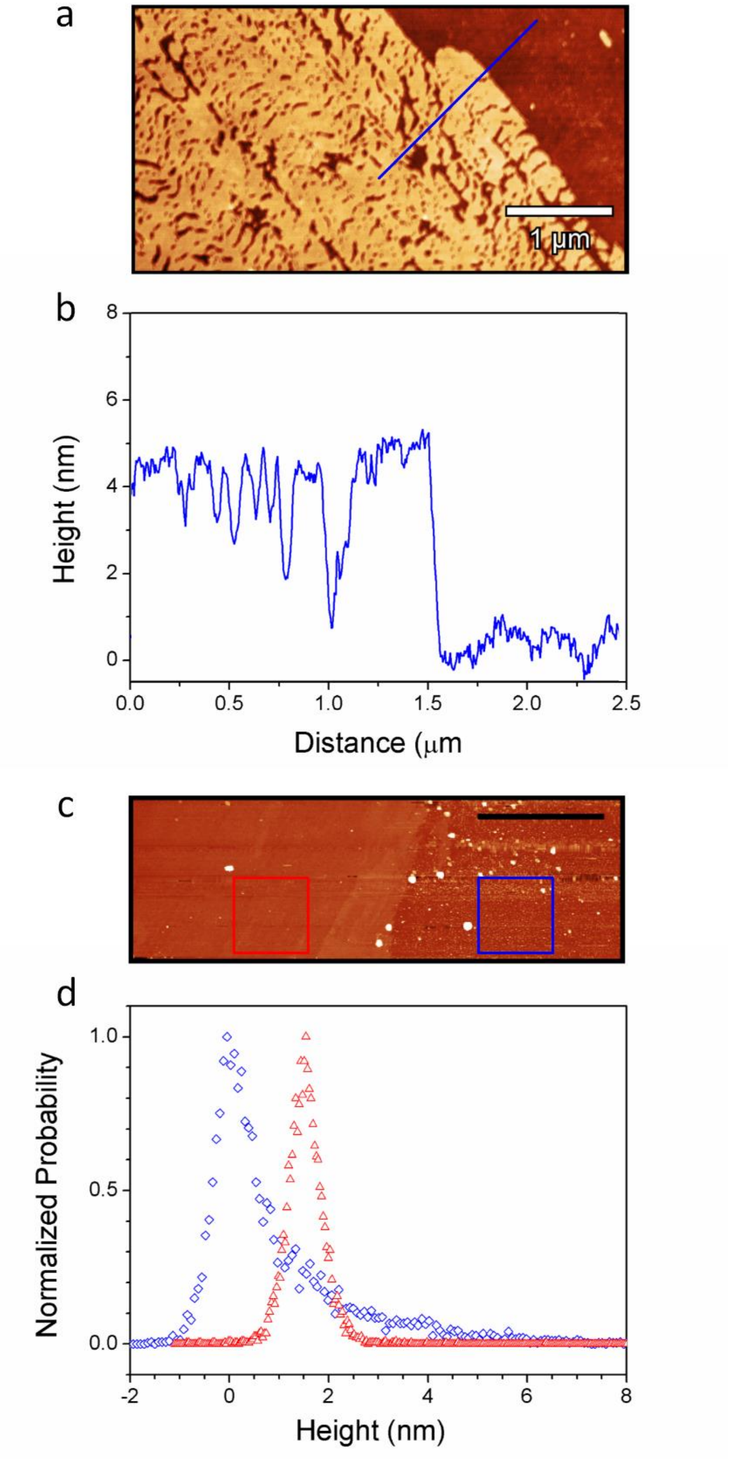

Supplementary Figure 1: (a) AFM image of cracked layer of gallium oxide and corresponding profile (b). As the liquid gallium/gallium oxide is printed, a scattered oxide layer appears with a strong adherence on the substrate. This cracked thin film of flat gallium oxide nano-flakes allows for the easy removal of the liquid metal by scraping with a slab of PDMS. This process is implementable even as the liquid metal is printed fresh because of the rapid formation of the oxide layer. (c) AFM image of 2D GaS film and statistical distribution of the measured pixel heights (d) of the substrate and film within the areas in the blue and red squares, respectively. Scale bar in (c) is 5 µm. The statistical distribution the 2D GaS shows that the film is highly continuous and the height distribution of the substrate (blue) and the film (red) are similar for values below than the mean height. However, above the mean, the distribution is extended further to the higher values for the substrate measurement leading to the skewed appearance. This is due to the hydrophobic nature of the substrate which leads to the deposition of some particles on its surface. In comparison, the surface of 2D GaS is much cleaner than the substrate, indicating controlled film growth. The standard deviation for the 2D GaS film is 0.137 nm which is associated with a continuous deposited films and the absence of nano or sub-micron size cracks as well as the absence of regions of overlapping growth that could occur at domain boundaries. Furthermore, there is no contribution from the substrate level (0 nm) in the red pattern attesting the absence of cracks and holes even further.

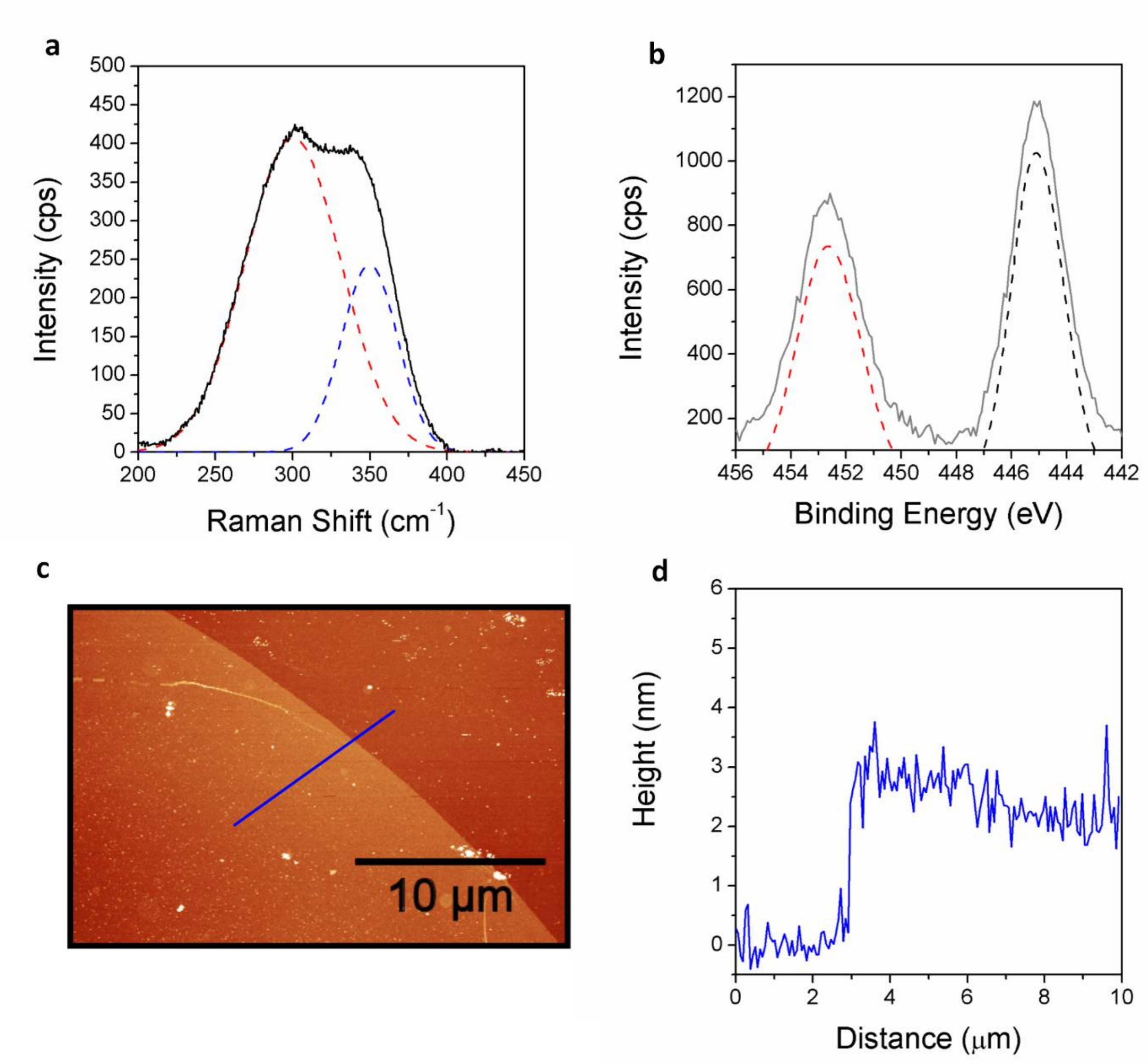

Supplementary Figure 2: Analysis of 2D In_2S_3 synthesised via printing of liquid indium. (a) Raman spectrum, (b) Indium 3d region of XPS and (c) AFM image including profile (d) along the blue line shown in (c). As can be seen, In_2S_3 film is smooth and continuous in a relatively large area range. The thickness of the 2D In_2S_3 is on average 2.6 nm, which is about three times the fundamental plane thickness (0.9 nm) of this material.

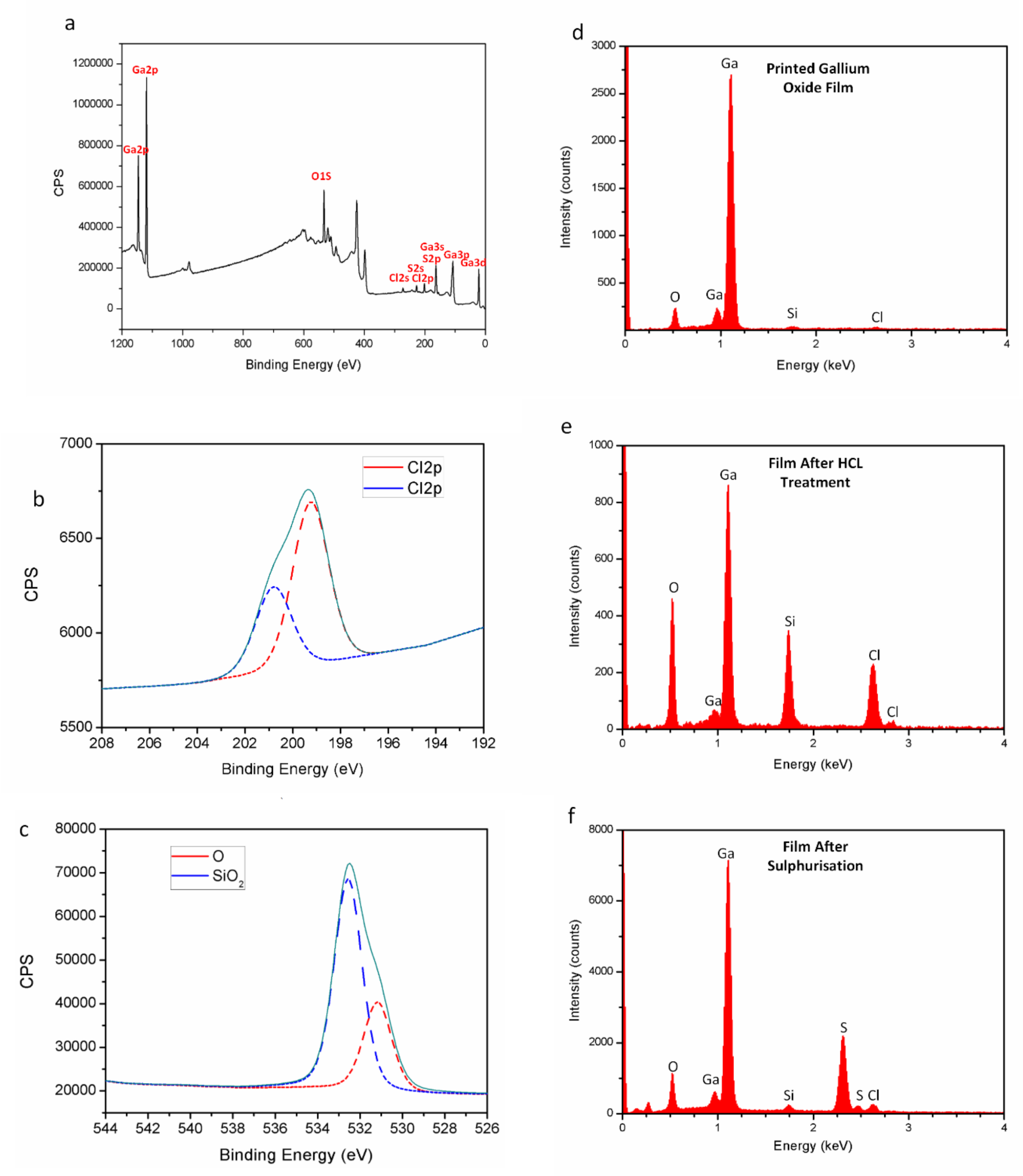

Supplementary Figure 3: (a-c) XPS study of GaS film displaying (a) survey spectrum, (b) Cl 2*p* and (c) O 1*s* regions of the spectrum. The O 1*s* range of the XPS spectrum demonstrates a significant contribution from SiO_2 (532.65 eV) while there are other minor contributions from other oxides including gallium-oxides[1]. The Cl 2*p* range of the XPS spectra also depicts some residual Cl. The intensity of this peak however (~1000 CPS above baseline) is almost negligible when compared to that of Ga or S peaks. (d-f) EDX of printed 2D GaO_x film (d), the film after treatment with HCl (e) and after sulphurisation (f).

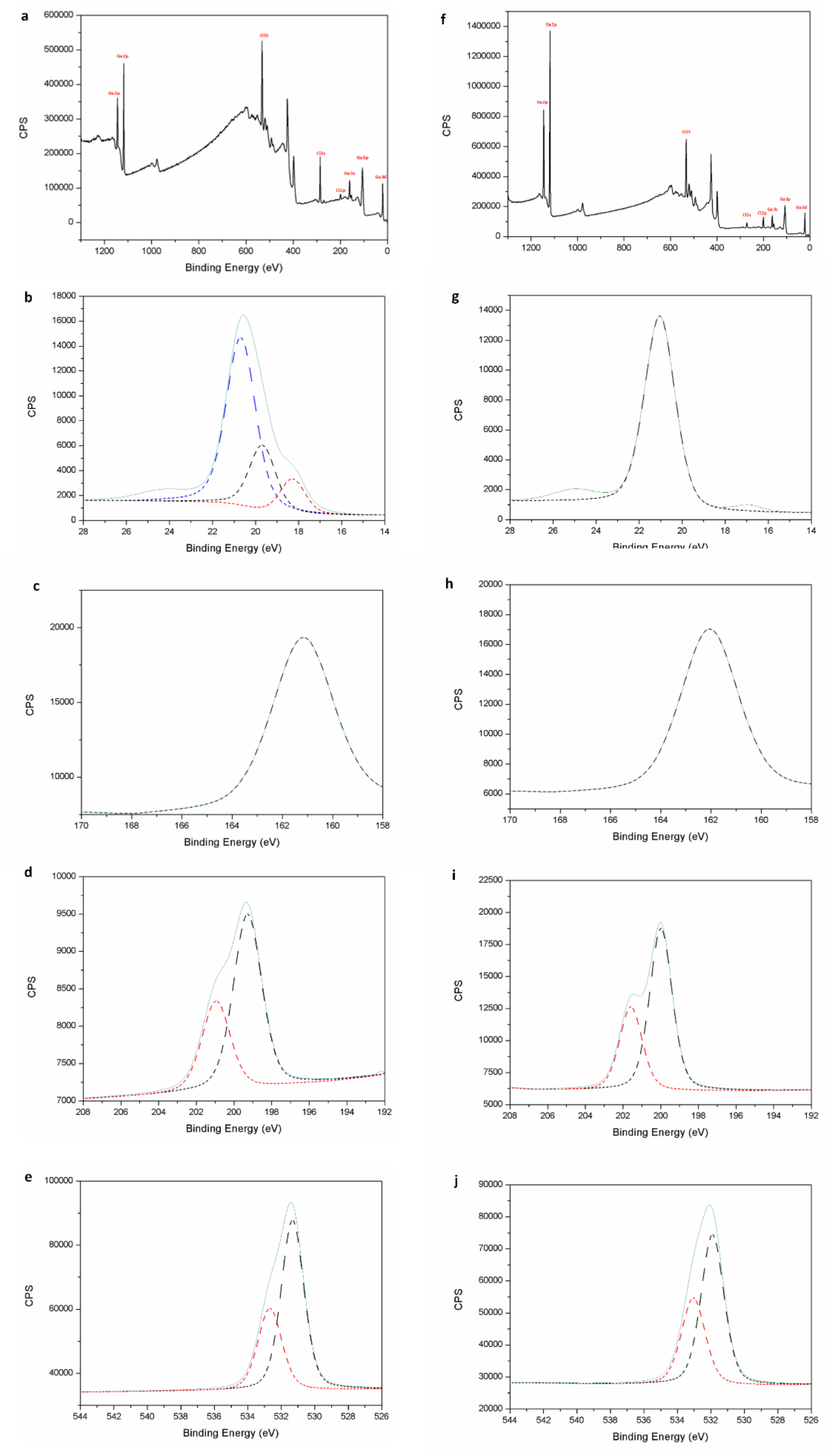

Supplementary Figure 4: XPS patterns of the printed metal oxide film before (a-e) and after (f-j) treatment with HCl vapour. (a & f) Survey, (b & g) Ga 3*d*, (c & h) Ga 3*s*, (d & i) Cl 2*p* and (e & j) O 1*s* regions.

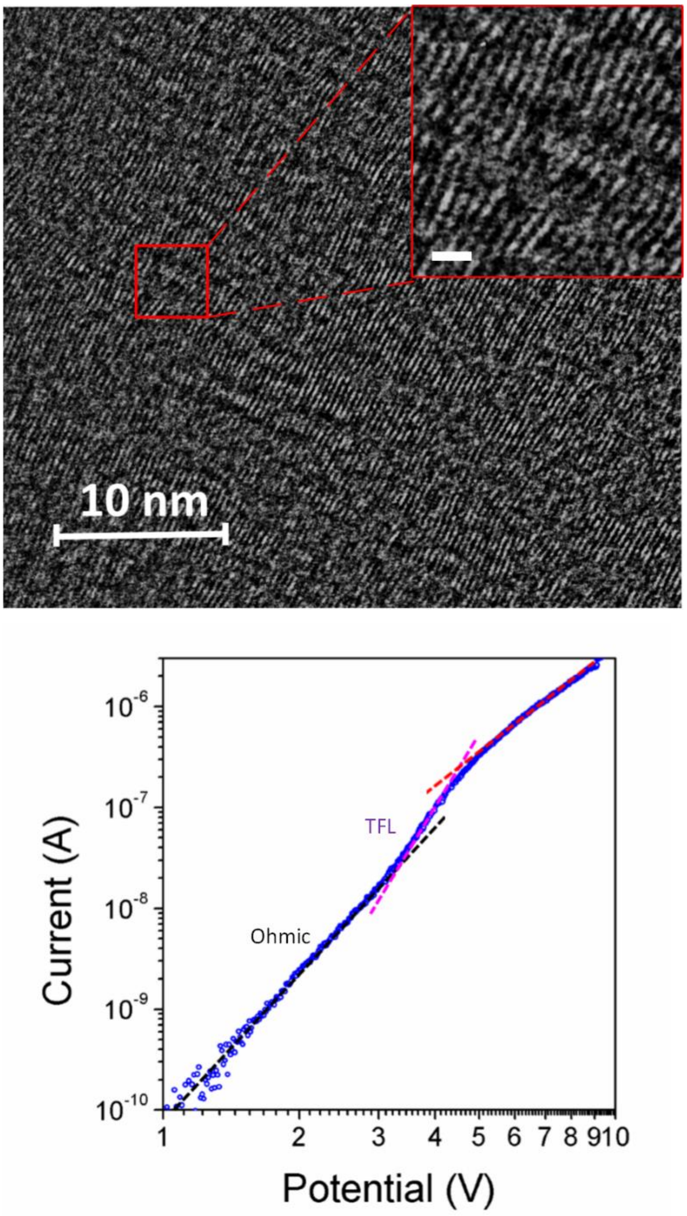

Supplementary Figure 5 (a) HRTEM of a GaS flake scratched from the printed film. Structural defects are visible in the crystal, which are mainly ascribed to lattice dislocations (example in the red square). The dislocation points introduce strain which cause bent lattice planes around the dislocation points[2]. Inset: magnified image highlighting the dislocation of the crystal lattice. From analysing the dislocations seen in many HRTEM images, we calculate a defect density of ~6.25×10^{11} cm^{-2}. (b) Space charge limited current (SCLC) measurement of 2D GaS. Ohmic and trap filled limited (TFL) regions are marked with their respective colours. Using the threshold voltage of the TFL region and the method as outlined by Shi *et al*[3], we determine a trap density of ~2.85×10^{11} cm^{-2} which is in agreement with our determined defect density from HRTEM.

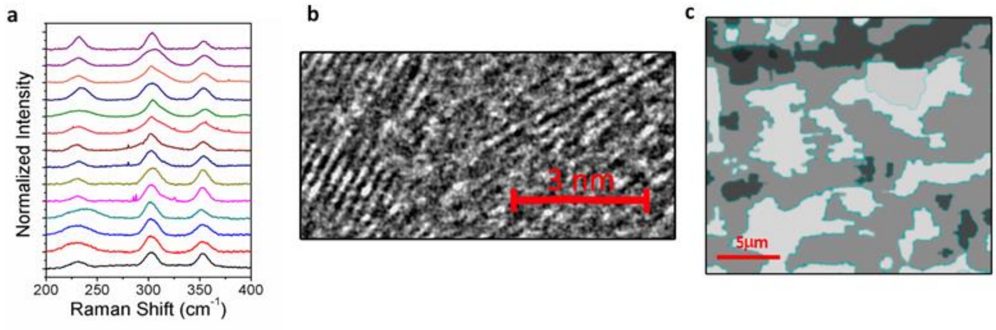

Supplementary Figure 6: (a) Raman spectra of 2D GaS spaced at approximately 1 mm intervals (much wider area than presented in figure 4). The relative intensity of the E^1_{2g} peak only slightly varies slightly when macroscopic distances of millimetres are investigated, demonstrating the strong consistency of the bilayer GaS across a large surface area. (b & c) Analysis of crystal grains of the printed 2D GaS film featuring (b) a HRTEM image of a grain boundary and (c) a map of grain boundaries determined via polarised confocal PL as has been previously demonstrated by Pan *et al*[4]. It can be seen from (b) that the boundaries are laterally connected with no overlap, however there are significant lattice defects around the boundaries. Furthermore, from (c) the domain sizes are seen to range from ~1 µm^2 to 10’s of µm^2. Different shades of grey indicate areas that luminesce at different polarisation angles which are associated with crystal domains and their lattice orientation within the 2D GaS layer.

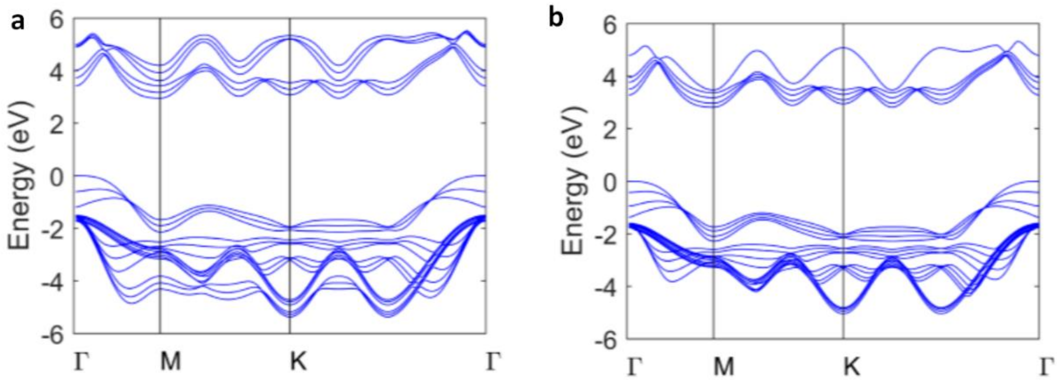

Supplementary Figure 7: Hybrid DFT computation of the band structures in (a) tri-layer, and (b) four-layer GaS.

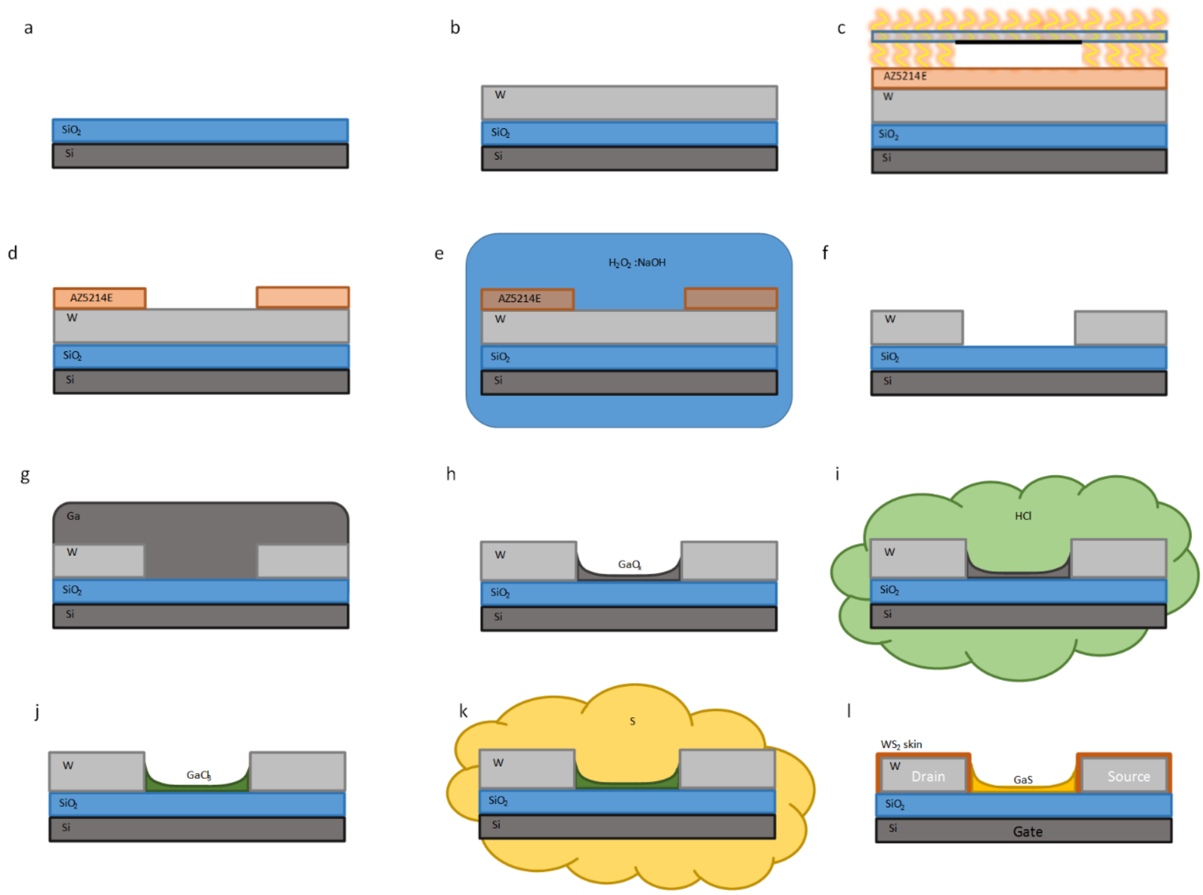

Supplementary Figure 8: Schematic representation for device fabrication. (a) Clean SiO_2/Si substrate. (b) Tungsten (W) film e-beam deposited onto the substrate. (c) Photoresist is deposited and then exposed through a photolithography mask. (d) Remaining photoresist after image reversal and developing. (e) Immersion of the of the unprotected W in the etchant. (f) Sample after etching and lift-off. (g) Deposition of liquid Ga. (h) Formation of 2D GaO_x between electrodes after Ga is removed. (i) Exposure to HCl vapour. (j) $GaCl_3$ film between electrodes. (k) Exposure to S vapour. (l) Final device. Extra photolithography steps can also be included as presented in Figure 1.

Supplementary Table 1: Comparison of established methods for deposition of 2D materials.

	Temperature (°C)	Time (hours)	Materials	Lateral dimensions	Comments	References
Atomic layer deposition (ALD)	300[5]	~2[5]	MoS_2[5]	µm's[5]	Requires annealing at 800°C[5]	[5] Tan *et al* 2014
Chemical vapour deposition (CVD)	800[6], 850[7]	~2[6], ~1[7]	WS_2[6], MoS_2[7]	µm's[6,7]		[6] Elias *et al* 2013<br>[7]Jeon *et al* 2015
Metalorganic CVD	550[8]	~26[8]	MoS_2[8]	µm's-mm's[8]		[8] Kang *et al* 2015
Thermolysis	1000[9]	1.5[9]	MoS_2[9]	mm's[9]		[9] Liu *et al* 2012
Mechanical Exfoliation			GaS[10], MoS_2[11], WSe_2[11]	µm's-10s µm[10,11]	Difficult to create reproducibility	[10] Late *et al* 2012<br>[11] Li *et al* 2015
Liquid phase epitaxy (LPE)		~9[1]	GaS[1], MoS_2[12], WS_2[12], h-BN[12]	nm's- 100s nm[1], 10s nm-µm's[12]		[1] Harvey *et al* 2015.<br>[12] Coleman *et al* 2011
This work	300	~2	GaS, In_2S_3	mm's		

References

1 Harvey, A. *et al.* Preparation of Gallium Sulfide Nanosheets by Liquid Exfoliation and Their Application As Hydrogen Evolution Catalysts. *Chem. Mater.* **27**, 3483-3493 (2015).

2 Mayrhofer, P. H., Mitterer, C., Hultman, L. & Clemens, H. Microstructural design of hard coatings. *Prog. Mater. Sci.* **51**, 1032-1114 (2006).

3 Shi, D. *et al.* Low trap-state density and long carrier diffusion in organolead trihalide perovskite single crystals. *Science* **347**, 519-522 (2015).

4 Pan, Z. *et al.* Polarization-resolved spectroscopy imaging of grain boundaries and optical excitations in crystalline organic thin films. *Nature Comm.* **6**, 8201 (2015).

5 Tan, L. K. *et al.* Atomic layer deposition of a MoS2 film. *Nanoscale* **6**, 10584-10588 (2014).

6 Elias, A. L. *et al.* Controlled synthesis and transfer of large-area WS2 sheets: from single layer to few layers. *Acs Nano* **7**, 5235-5242 (2013).

7 Jeon, J. *et al.* Layer-controlled CVD growth of large-area two-dimensional MoS 2 films. *Nanoscale* **7**, 1688-1695 (2015).

8 Kang, K. *et al.* High-mobility three-atom-thick semiconducting films with wafer-scale homogeneity. *Nature* **520**, 656-660 (2015).

9 Liu, K.-K. *et al.* Growth of large-area and highly crystalline MoS2 thin layers on insulating substrates. *Nano letters* **12**, 1538-1544 (2012).

10 Late, D. J. *et al.* GaS and GaSe ultrathin layer transistors. *Adv. Mater.* **24**, 3549-3554 (2012).

11 Li, H., Wu, J., Yin, Z. & Zhang, H. Preparation and applications of mechanically exfoliated single-layer and multilayer MoS2 and WSe2 nanosheets. *Acc. Chem. Res.* **47**, 1067-1075 (2014).

12 Coleman, J. N. *et al.* Two-dimensional nanosheets produced by liquid exfoliation of layered materials. *Science* **331**, 568-571 (2011).
